# A Mitogen-Activated Protein Kinase Tmk3 Participates in High Osmolarity Resistance, Cell Wall Integrity Maintenance and Cellulase Production Regulation in *Trichoderma reesei*

**DOI:** 10.1371/journal.pone.0072189

**Published:** 2013-08-26

**Authors:** Mingyu Wang, Qiushuang Zhao, Jinghua Yang, Baojie Jiang, Fangzhong Wang, Kuimei Liu, Xu Fang

**Affiliations:** 1 State Key Laboratory of Microbial Technology, Shandong University, Jinan, China; 2 National Glycoengineering Research Center, Shandong University, Jinan, China; 3 Department of Surgery, VA Boston Healthcare System, Boston University, Boston, Massachusetts, United States of America; 4 Cancer Research Center, Shandong University School of Medicine, Jinan, China; University of Nebraska, United States of America

## Abstract

The mitogen-activated protein kinase (MAPK) pathways are important signal transduction pathways conserved in essentially all eukaryotes, but haven't been subjected to functional studies in the most important cellulase-producing filamentous fungus *Trichoderma reesei*. Previous reports suggested the presence of three MAPKs in *T. reesei*: Tmk1, Tmk2, and Tmk3. By exploring the phenotypic features of *T. reesei Δtmk3*, we first showed elevated NaCl sensitivity and repressed transcription of genes involved in glycerol/trehalose biosynthesis under higher osmolarity, suggesting Tmk3 participates in high osmolarity resistance via derepression of genes involved in osmotic stabilizer biosynthesis. We also showed significant downregulation of genes encoding chitin synthases and a β-1,3-glucan synthase, decreased chitin content, ‘budded’ hyphal appearance typical to cell wall defective strains, and increased sensitivity to calcofluor white/Congo red in the *tmk3* deficient strain, suggesting Tmk3 is involved in cell wall integrity maintenance in *T. reesei*. We further observed the decrease of cellulase transcription and production in *T. reesei Δtmk3* during submerged cultivation, as well as the presence of MAPK phosphorylation sites on known transcription factors involved in cellulase regulation, suggesting Tmk3 is also involved in the regulation of cellulase production. Finally, the expression of cell wall integrity related genes, the expression of cellulase coding genes, cellulase production and biomass accumulation were compared between *T. reesei Δtmk3* grown in solid state media and submerged media, showing a strong restoration effect in solid state media from defects resulted from *tmk3* deletion. These results showed novel physiological processes that fungal Hog1-type MAPKs are involved in, and present the first experimental investigation of MAPK signaling pathways in *T. reesei*. Our observations on the restoration effect during solid state cultivation suggest that *T. reesei* is evolved to favor solid state growth, bringing up the proposal that the submerged condition normally used during investigations on fungal physiology might be misleading.

## Introduction

The systematic risk inherent to a fossil fuel based economy has drawn worldwide attention, leading to the proposal of replacing these non-renewable energy sources with renewable energy sources including energy from renewable biomass. One such biomass is lignocellulose, which is abundantly available as agricultural or forestry wastes. Conversion of lignocellulose to energy sources that are compatible to modern industry is a complicated process, in which the most important step is the saccharification of lignocellulose, catalyzed by cellulases produced from filamentous fungi [1]. The most widely used and important cellulase hyper-producing organism is *Trichoderma reesei*. Physiological investigations of this so-called industrial cellulase-producing ‘workhorse’ are therefore essential in both scientific and technological regards [2].

Cells sense their surrounding environments and react to external signals via signal transduction pathways. Among all known signal transduction pathways, the mitogen-activated protein kinase (MAPK) pathway is ubiquitous in almost all eukaryotic species and is one of the most well characterized pathways [3]. This pathway features a signal relay cascade in which three kinases are involved: the MAPK kinase kinase (MAPKKK), the MAPK kinase (MAPKK) and MAPK [4]. MAPK further phosphorylates downstream elements involved in the regulation of physiological activities. The most extensive research of these pathways was carried out in *Saccharomyces cerevisiae*, in which six MAPK pathways were identified [5]. In filamentous fungi, three major classes of MAPKs are present, respectively homologous to yeast Hog1p, Slt2p, and Fus3p. These MAPKs have been shown to function in a variety of physiological processes such as fruiting body development [6], polarized growth [7], biosynthesis [8], conidiation [9], pathogenicity [10], circadian rhythmicity [11], stress response [12], [13], protein production [9], [14] and cell wall integrity maintenance [15].

The HOG (high-osmolarity glycerol) pathway in *S. cerevisiae* is involved in combating high osmolarity by activating genes required for viability under hypertonic stress, which include genes in the glycerol synthesis pathway encoding glycerol-3-phosphate dehydrogenase (*GPD1*) and glycerol–3-phosphatase (*GPP2*) [16], [17]. Governing this pathway are kinases in the Ste11p/Ssk2p/Ssk22p-Pbs2p-Hog1p (MAPKKK-MAPKK-MAPK) pathway [18]. In filamentous fungi, this function of the Hog1p pathway seems to be generally conserved. Hog1p homologues were shown to be involved in high osmolarity resistance in *Aspergillus nidulans* [19], *Magnaporthe grisea* [10], *Cryphonectria parasitica* [13], *Neurospora crassa* [20], *Trichoderma harzianum* [21], and *Aspergillus fumigatus* [22].

Investigations carried out on Slt2p homologues in filamentous fungi suggested the involvement of this MAPK in the maintenance of cell wall integrity in *A. fumigatus* [12], [15], *Magnaporthe oryzae* [23], *Trichoderma virens* [24], *Claviceps purpurea* and *Fusarium graminearum* [25], but not *Mycosphaerella graminicola* (*Mgslt2*) and *Botrytis cinerea* [26]. The involvement of a gene in cell wall integrity maintenance is generally examined by testing for hypersensitivity in the gene deletion strain to cell wall lysing enzymes or cell wall interfering compounds such as calcofluor white (CFW) and Congo red (CR). Interestingly, the deletion of *hog1* homologues in *T. harzianum* and *A. fumigatus* showed unchanged or even increased resistance to CFW and CR, suggesting the hypersensitivity to improved osmolarity is unrelated to weakening of the cell wall [21], [27].

In filamentous fungi, yeast Fus3p homologues are involved in quite diverse processes, one of which is the production of glycoside hydrolases. Cellulase and chitinase production was upregulated in the *tmkA* (a *fus3* homologue) deletion strain of *T. virens* [9]. Chitinase production was improved in the *tmk1* (a *fus3* homologue) knockout strain of *Trichoderma atroviride* [14]. The expression levels of *N*-acetylglucosaminidase- and chitinase-encoding genes increased when *tvk1* (a *fus3* homologue) was disrupted in *T. virens* [28]. This phenomenon is of particular interest in cellulase-producing filamentous fungi like *Trichoderma* and *Aspergillus* species, as they are well-known industrial cellulase hyper-producing strains, out of which *T. reesei* is the most widely studied and used.

*T. reesei* (syn. *Hypocrea jecorina*) was first isolated on the Solomon Islands during World War II, and has received considerable improvements over the last seventy years for industrial applications [29]. It has turned a paradigm for the investigations of cellulases, hemicellulases, and the molecular mechanisms underlying their synthesis and regulation [30]. However, knowledge on the signal transduction cascades in this fungus is limited to a few aspects: 1) light-modulated cellulase production mediated by G proteins (Gna1 and Gna3), a PAS/LOV domain protein ENVOY and cAMP-dependent protein kinase A signaling [31]–[34]; 2) regulation of the sexual development process [35]; 3) cellulose- and cAMP-independent modulation of cellulase production mediated by Ras GTPase TrRas2 [36]. Relatively little is known in other well-known signal transduction pathways such as MAPK pathways, Ca^2+^-signaling pathways and factors such as casein kinase II, germinal center kinases and protein kinase C.

*In silico* reconstruction of the MAPK signal transduction cascade in *T. reesei* identified three distinct pathways, in which three putative MAPKs are involved: the yeast Fus3p homologue Tmk1, Slt2p homologue Tmk2, and the Hog1p homologue Tmk3 [37]. None of these MAPKs have been studied in *T. reesei* yet. In this study, by characterizing the properties of the *T. reesei Δtmk3* deletion strain, we attempt to identify the role of Tmk3 in *T. reesei*, particularly in cell wall integrity maintenance and cellulase production that have not been identified for Hog1-type MAPKs in other fungal species. Further comparison between submerged and solid state growth leads to the finding of novel, interesting restoration effects during solid state cultivation. These studies are the first research done on MAPKs in *T. reesei*, leading up to further in-depth understanding of the regulatory mechanisms of this well-known industrial cellulase hyper-producing workhorse.

## Materials and Methods

### Strain and chemicals

*T. reesei Δku70* strain, derived from the QM9414 uridine auxotrophic *pyr4*-negative strain TU-6 (ATCC MYA-256), was used as the high transformation efficiency parent strain for the gene deletion experiment, as is previously reported [38]. Uridine and sorbitol were purchased from Sangon Biotech Co., Ltd. (Shanghai, China). Calcofluor white (CFW), *p*-nitrophenyl-β-_D_-glucopyranoside (*p*NPG), *p*-nitrophenyl-β-_D_-cellobioside (*p*NPC), carboxymethylcellulose (CMC), cytohelicase from *Helix pomatia* and chitinase from *Streptomyces griseus* were purchased from Sigma-Aldrich Corporation (St. Louis, MO, US). *p*-nitrophenyl-β-_D_- xylopyranoside (*p*NPX) was purchased from Tokyo Chemical Industry Co., Ltd. (Tokyo, Japan). Congo red was purchased from Tianjin Damao Chemical Reagent Factory (Tianjin, China). Wheat bran was kindly provided by Longlive Bio-Technology Co., Ltd., Yucheng, Shandong, China. All other chemicals were purchased from Sinopharm Chemical Reagent Co., Ltd. (Shanghai, China).

### Phylogenetic analysis

Phylogenetic tree construction and protein sequence comparison were carried out using the Clustal X2 software [39].

### Construction of *tmk3* deletion strain

Construction of *T. reesei Δtmk3* was carried out essentially as previously described [38], using the *pyr4* selection marker which restores uridine biosynthesis capabilities in the *pyr4*-deficient parent strain. Amplification of *tmk3* from *T. reesei* genome was carried out using Kod FX high fidelity enzymes (TOYOBO CO. LTD. Osaka, Japan).

### Southern blotting analysis

Southern blotting experiment was used to confirm whether *T. reesei Δtmk3* was successfully constructed. Genomic DNA extracted from *T. reesei Δku70* or *Δtmk3* strain was digested using HindIII prior to hybridization. Detection of probe-hybridized DNA fragment was carried out using the DIG High Prime DNA Labeling and Detection Starter Kit I (Roche Diagnostics, Mannheim, Germany).

### Submerged and solid state growth

Minimal media solution containing 0.5% NH_4_SO_4_, 0.06% MgSO_4_, 1.5% KH_2_PO_4_, 0.08% CaCl_2_, 0.00005% FeSO_4_·7H_2_O, 0.00016% MnSO_4_·H_2_O, 0.00014% ZnSO_4_·7H_2_O and 0.00002% CoCl_2_ was first prepared. Spores of *T. reesei Δku70* or *Δtmk3* strain were harvested after 6 days of growth, and counted using a hemacytometer. For submerged growth, approximately 10^7^ spores were inoculated in submerged media containing 2 g avicel, 2 g wheat bran and 100 ml minimal media solutions. The media for growth of *T. reesei Δku70* contain 0.1% uridine. The growth of 100 ml submerged cultures took place in 500 ml flasks at 30°C in a rotary shaker (Model SKY-1112B, Shanghai Sukun Ltd., Shanghai, China) rotated at 200 rpm. Solid state media contain 6 ml minimal media solution, 2 g avicel and 2 g wheat bran. Approximately 10^7^ spores were inoculated to glass plates containing 10 g solid state media. The plates were subsequently incubated in an incubator (Model MJX-250, Ningbo Jiangnan Instrument Factory, Ningbo, China) at 30°C without shaking.

### Phenotypic analysis

Approximately 10^6^ conidiospores of *T. reesei Δku70* or *Δtmk3* strain were inoculated on minimal media (MM) plates containing glucose, starch, sucrose, lactose or glycerol, as well as PDA plates. The plates were incubated in an incubator (Model MJX-250, Ningbo Jiangnan Instrument Factory, Ningbo, China) at 30°C for 4 days. Double-layer avicel plates were prepared by first casting a MM agarose bottom layer containing no carbon sources, and a second MM agarose top layer containing 1% avicel. Approximately 10^6^ conidiospores of *T. reesei Δku70* or *Δtmk3* strain were inoculated on the plates, which were incubated at 30°C for 4 days prior to examination.

Images of *T. reesei Δku70* or *Δtmk3* hyphae were taken with a bright field microscope (Nikon eclipse E100, 400 fold magnification) from cultures grown in submerged minimal media containing glucose as the carbon source.

### NaCl, CFW and CR sensitivity assays

Approximately 10^6^ spores of *T. reesei Δku70* or *Δtmk3* strain were inoculated on PDA plates containing various concentrations of NaCl, CFW and CR for sensitivity tests on these chemicals. These plates were subsequently incubated in an incubator (Model MJX-250, Ningbo Jiangnan Instrument Factory, Ningbo, China) at 30°C for 3 days. The dosages of NaCl used in this assay are respectively 0, 0.3, 0.5, 0.7, 0.9, 1.1 M. The dosages of CFW used in this assay are respectively 0, 10, 20, 30, 40 mg/L. The dosages of CR used in this assay are respectively 0, 75, 125, 150, 175, 200 mg/L. The diameters of colonies on CR- and CFW-containing plates were measured for comparison between *T. reesei Δku70* and *Δtmk3*. Three individual replicates of each experiment were performed.

### Biochemical assays

The ATP level was assayed using the Checklite 250 plus ATP kit (Kikkoman Biochemifa Company, Minato-ku, Japan). The concentration of ATP indicates the average ATP concentration in cultures. Five individual replicates were carried out for ATP level analysis in solid state cultures. Three individual replicates were carried out for ATP level analysis in submerged cultures.

The DNA content of a *T. reesei Δku70* or *Δtmk3* culture was also used as a measure of biomass. To measure the DNA content, solid state and submerged cultures were first prepared as is described in this work. For submerged cultures, 1 ml of the culture was drawn from flasks for DNA content determination. For solid state cultures, 25 ml of distilled water was used to suspend the cultures, and 1 ml of suspended culture was drawn for DNA content determination. The cultures were subsequently diluted 5-fold in 15 ml centrifuge tubes, and were subjected to a brief centrifugation to remove supernatant. One milliliter of 10% trichloroacetic acid (TCA) was then added to the tube, thoroughly mixed, and incubated on ice for 3 minutes. The tube was boiled in a water bath (Shanghai Jinghong Laboratory Instrument Co. Ltd., Shanghai, China) for 30 minutes, followed by centrifugation at 10,000 rpm for 10 minutes. Absorbance at 260 nm in the supernatant was subsequently measured using a UV-visible spectrophotometer (Model 2802, UNICO, Dayton, NJ, US). The total DNA content in the cultures was calculated from A_260_. Three individual replicates were carried out for each assay.

Protein concentration was determined using the Lowry method [40]. Filter paperase activity (FPA) was assayed following previously published protocols [41]. Cellobiohydrolase, endoglucanase, β-glucosidase and β-xylosidase activities were respectively assayed by abilities to hydrolyze *p*NPC, CMC, *p*NPG and *p*NPX following published protocols [42]. Six replicates were performed to determine extracellular protein concentrations, *p*NPCase, CMCase, *p*NPGase and *p*NPXase activities in solid state cultures. Three replicates were performed to determine extracellular protein concentrations, *p*NPCase, CMCase, *p*NPGase and *p*NPXase activities in submerged cultures.

### Chitin content assay

Chitin contents of *T. reesei Δku70* and *T. reesei Δtmk3* were assayed similarly to previous reports [43], [44]. Approximately 10^6^ spores of *T. reesei Δku70* or *Δtmk3* strain were inoculated in submerged media containing 2% glucose. The mycelia were harvested after 3 days of growth, and subsequently dried by heating at 105°C for 4 hours in an oven (Model DHG-9030, Shanghai Jinghong Laboratory Instrument Co. Ltd., Shanghai, China) for the determination of dry cell weight (DCW). Sixty mg of mycelia (14 mg DCW) of both *T. reesei Δku70* or *Δtmk3* strain were heated at 80°C for 90 minutes in 1 ml of 6% KOH using a water bath (Shanghai Jinghong Laboratory Instrument Co. Ltd., Shanghai, China), followed by addition of 0.1 ml glacial acetic acid. The treated mycelia were further centrifuged in a microcentrifuge (Centrifuge 5415R, Eppendorf, Hamburg, Germany) at 13,000 rpm for 10 minutes. The pellet was suspended in 0.5 ml phosphate buffer (pH 6.3) and digested using 0.1 U chitinase by incubation at 37°C for 1 hour. The reaction system was centrifuged again at 13,000 rpm for 10 minutes. Zero point two five mg of cytohelicase was subsequently added to 500 μl supernant, followed by incubation at 37°C for 1 hour. The amount of released *N*-acetylglucosamine was assayed according to previous reported procedures [44].

### Real-time PCR reactions and data manipulation

Total RNA was extracted from *T. reesei Δku70* or *Δtmk3* strain growing in solid state media and submerged media containing 2% avicel and 2% wheat bran for the examination of expression of cellulase-, hemicellulase-, chitin synthase- and β-1,3-glucan synthase-coding genes, as well as from *T. reesei Δku70* or *Δtmk3* strain growing in submerged media containing 2% glucose and 0 or 0.15 M NaCl for the examination of expression of glycerol-3-phosphate dehydrogenase- and α,α -trehalose-6-phosphate synthase-coding genes. cDNA was synthesized using PrimeScript RT reagent kit with gDNA erase (Perfect Real Time) from Takara Bio Inc. (Shiga, Japan).

Real-time PCR reactions were carried out on a LightCycler 480II Real-Time PCR system (Roche Applied Science, Mannheim, Germany) using SYBR Premix EX Taq^TM^ II (Takara Bio Inc., Shiga, Japan) as the dye. Three individual biological replicates and three individual technical replicates for each biological sample (a total of 9 replicates for each reaction) were carried out. The relative abundance of genes was calculated using the 2^−ΔΔCt^ method as previously described [45].

## Results and Discussion

### *tmk3* encodes a Hog1-type MAPK in *T. reesei*

Phylogenetic analysis of Tmk3 from *T. reesei* and other previously characterized MAPKs showed Tmk3 apparently cluster with Hog1-type MAPKs from other species (Fig. S1), leading to the suggestion that *tmk3* likely encodes a Hog1 type MAPK. Sequence comparison between Tmk3 from *T. reesei* and Hog1 from *S. cerevisiae* showed a sequence identity of 66%, further supporting this suggestion (Fig. S2). It can therefore be concluded that Tmk3 is homologous to Hog1-type MAPKs, which function in high osmolarity resistance in *S. cerevisiae* and other filamentous fungi [3].

### Construction and growth patterns of *T. reesei Δtmk3*

**Figure 1:**
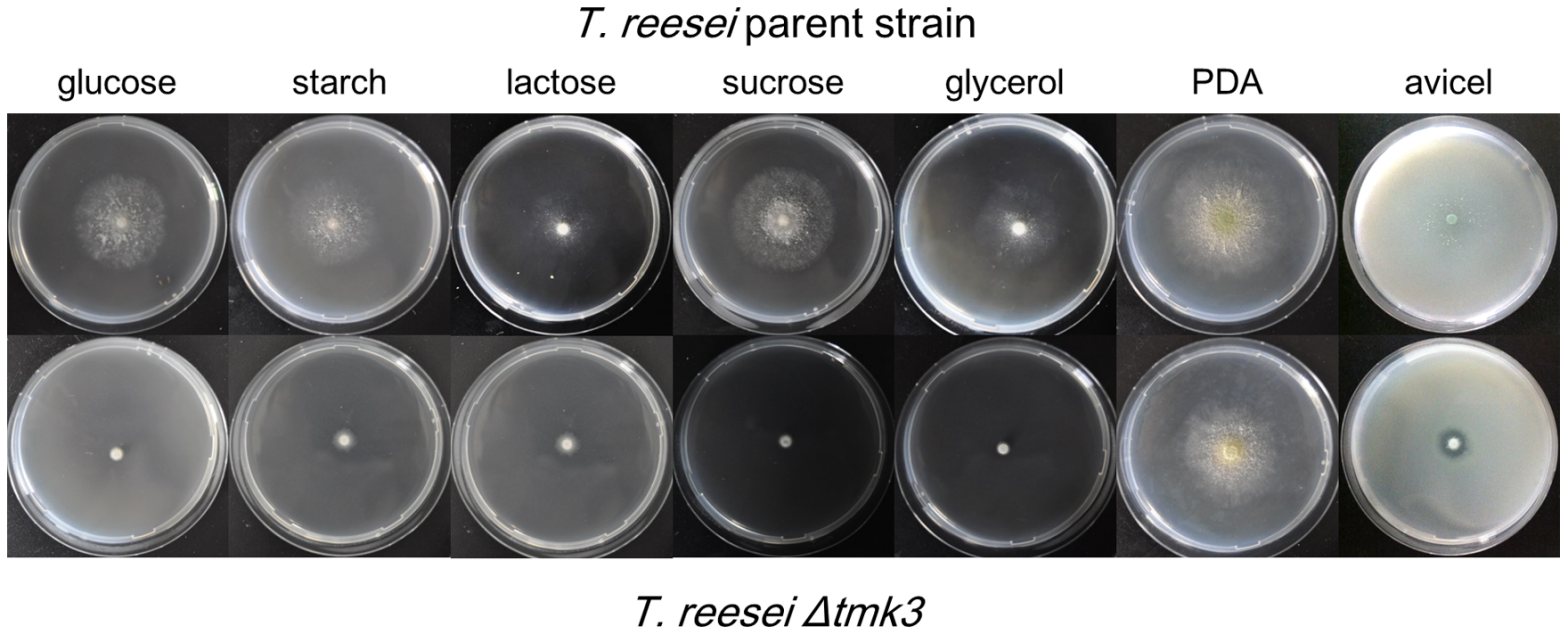
Growth of *T. reesei* parent strain and *T. reesei Δtmk3* on plates.

The *tmk3* deletion strain was constructed via homologous recombination using *T. reesei Δku70* as the parent strain (Fig. S3), in which the non-homologous end joining pathway was defective [38]. Examination of the growth of parent and *Δtmk3* strains on plates lead to several interesting findings: 1) The growth of *T. reesei Δtmk3* on each minimal media plate containing a tested carbon source (glucose, starch, lactose, sucrose, glycerol) is significantly worse than the parent strain; 2) Only slightly slower growth was observed when *T. reesei Δtmk3* was grown on complete media (PDA plates); 3) a significantly larger transparent zone was observed around the colony of *T. reesei Δtmk3* in comparison to the parent strain, when the two strains were grown on double layer plates containing avicel (Fig. 1).

The differences between the performance of *T. reesei Δtmk3* on minimal and complete media reflect damaged biosynthesis of certain critical compounds in the *tmk3*-deficient strain. The availability of these compounds in complete media partially compensates for the defects. Our results cannot specifically suggest which pathways are damaged, which remains to be elucidated in further studies.

The larger transparent zone around the colony on avicel-containing double layer plate is an indication of improved avicelase production in *T. reesei Δtmk3*. The term ‘avicelase’ here refers to the avicel-hydrolyzing enzymatic activities, which are primarily determined by cellobiohydrolase activities for two reasons: 1) cellobiohydrolase is the most abundant enzyme in secreted *T. reesei* cellulases [46]; 2) avicel has a highly crystalized cellulose structure and lacks the amorphous regions favored by endoglucanases. Although the contribution of other major cellulases cannot be excluded, the improved avicelase production in *T. reesei Δtmk3* can be interpreted primarily as the increase of cellobiohydrolase secretion.

**Figure 2:**
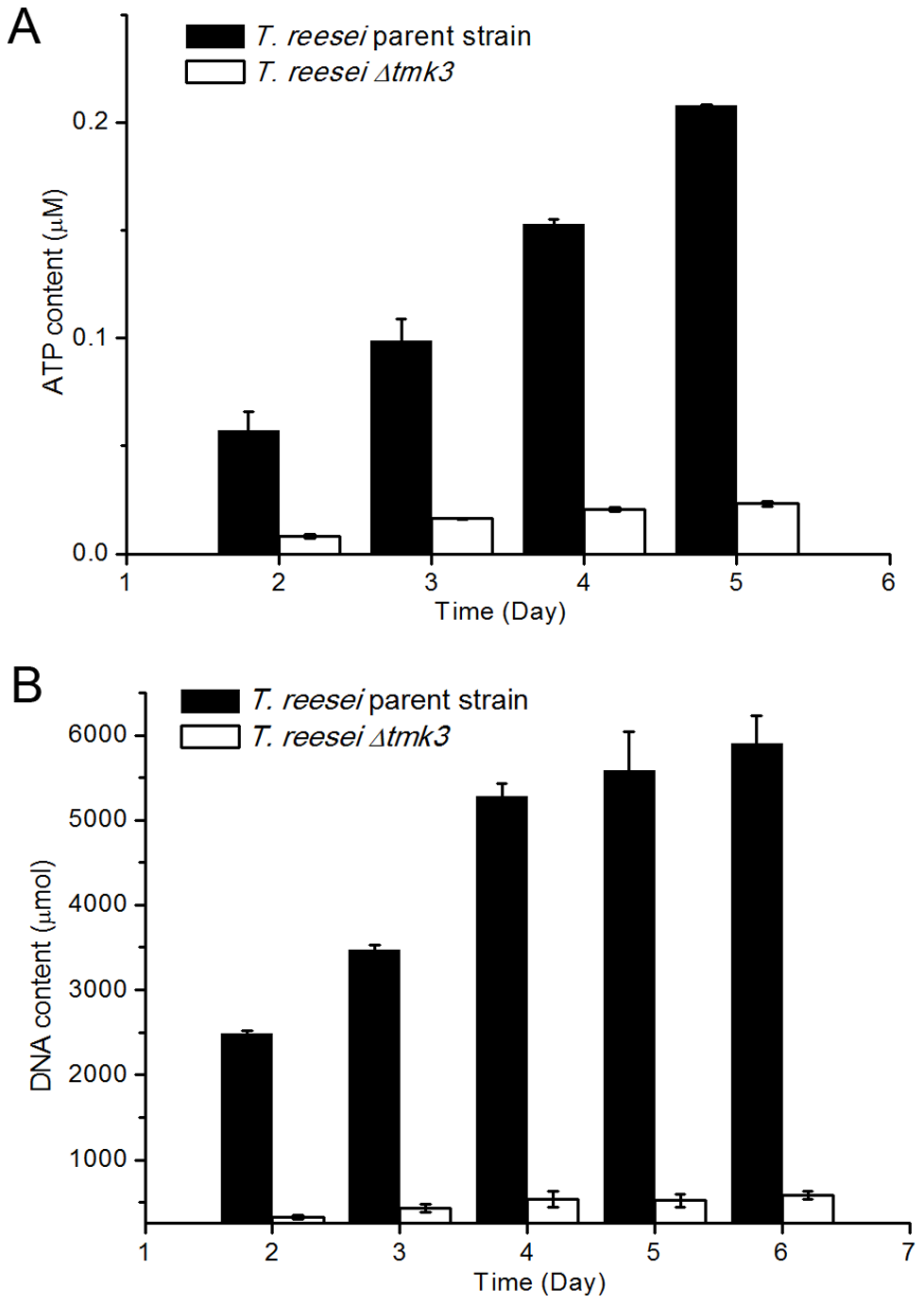
Biomass accumulations in submerged media. Panel A, biomass measured by ATP concentration in cultures; Panel B, biomass measured by total DNA content in cultures.

Growth of *T. reesei* parent and *Δtmk3* strains was also compared during submerged cultivation. The levels of ATP production and DNA content were used as measures of biomass accumulation. As is shown in Fig. 2, growth is significantly hampered in the *tmk3*-deficient strain, suggesting the important role Tmk3 plays in vegetative growth.

### Participation of Tmk3 in high osmolarity resistance via derepression of glycerol and trehalose synthesis genes

**Figure 3:**
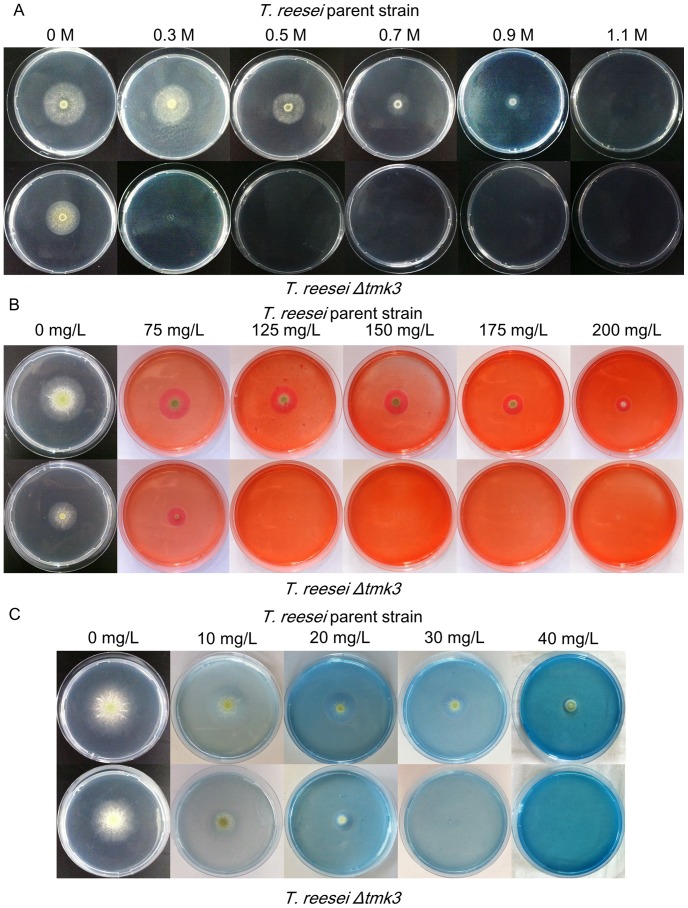
Test of sensitivity to NaCl, CR and CFW. Panel A, sensitivity to NaCl; Panel B, sensitivity to CR; Panel C, sensitivity to CFW.

We examined the tolerance of *T. reesei* parent and *Δtmk3* strains to elevated osmotic pressure by growing them on plates containing various concentrations of NaCl. It appears that *T. reesei Δtmk3* colonies ceased to develop at the NaCl level of 0.5 M, while the parent strain can still grow in the presence of 0.9 M NaCl (Fig. 3A). The apparent higher sensitivity to NaCl for the *tmk3* deletion strain is an indication of hampered tolerance to high osmolarity, which is indicative of a Hog1-like function for Tmk3.

**Figure 4:**
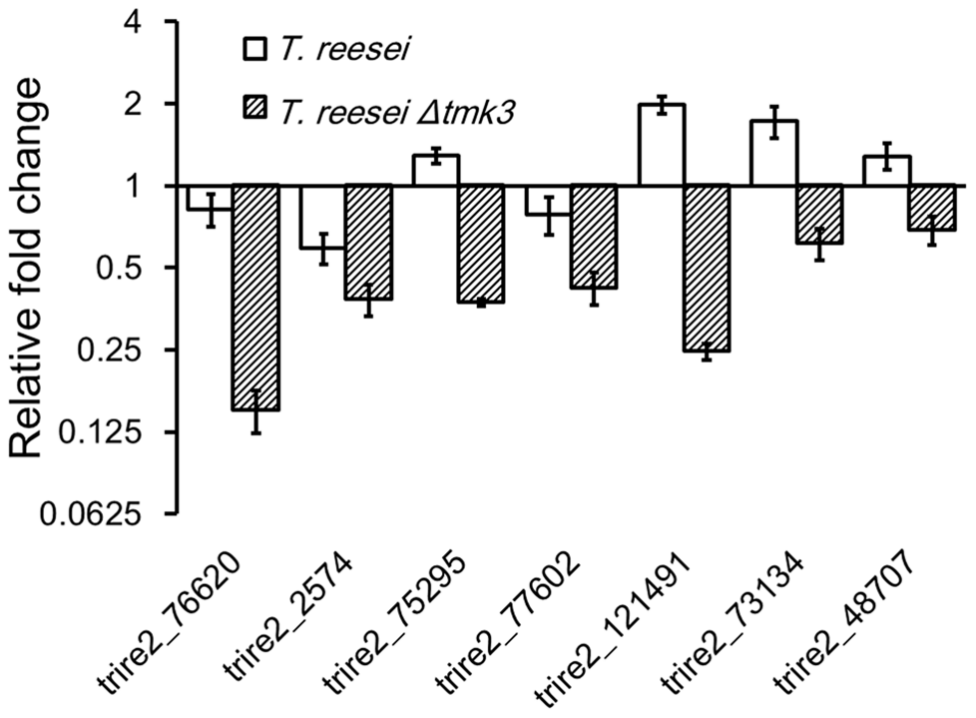
Response of transcriptional levels of glycerol and trehalose biosynthesis genes to elevated osmolarity. Y-axis indicates transcriptional abundance ratio between strains grown under 0.15 M NaCl and 0 M NaCl. Trire2_76620, Trire2_2574, glycerol-3-phosphate dehydrogenase-coding genes; Trire2_75295, Trire2_77602, Trire2_121491, Trire2_73134, Trire2_48707, α,α -trehalose-6-phosphate synthase-coding genes.

It was reported that glycerol and trehalose are involved in high osmolarity resistance by improving intracellular osmolarity in *S. cerevisiae* [16], [47]. In particular, *gpd1* that encodes a glycerol-3-phosphate dehydrogenase and functions in glycerol biosynthesis was upregulated during exposure to high osmolarity [16]. In *T. reesei*, we found two glycerol-3-phosphate dehydrogenase-coding genes functioning in glycerol biosynthesis (Trire2_76620 and Trire2_2574) and five α,α-trehalose-6-phosphate synthase-coding genes (Trire2_75295, Trire2_77602, Trire2_121491, Trire2_73134 and Trire2_48707) functioning in trehalose biosynthesis. Unlike in *S. cerevisiae*, the upregulation of these genes in response to improved osmolarity is not apparent in *T. reesei* parent strain (Fig. 4). In *T. reesei Δtmk3*, however, the expression levels of these genes are downregulated when exposed to 0.15 M NaCl (Fig. 4). These results suggest the Hog1-homologue Tmk3 functions in high osmolarity resistance in *T. reesei*, similarly to *S. cerevisiae*. The mechanism of this function, however, appears different: Hog1p of *S. cerevisiae* functions in stimulation of genes in the osmotic stabilizer biosynthesis; Tmk3 of *T. reesei* functions in derepression of genes in the osmotic stabilizer biosynthesis.

### Involvement of Tmk3 in cell wall structure integrity maintenance

The integrity of *T. reesei* parent and *Δtmk3* strains' cell wall was investigated by testing of sensitivity to cell wall interfering substances CFW and CR. On CFW- and CR-containing plates, *T. reesei Δtmk3* colonies are significantly smaller than those of the parent strain (Fig. 3B, 3C). *T. reesei Δtmk3* could not grow on plates containing 125 mg/L CR or 30 mg/L CFW, while the parent strain was able to tolerate at least 200 mg/L CR and 40 mg/L CFW. Analysis of colony diameters showed *T. reesei Δtmk3* is affected by CFW and CR more severely than *T. reesei* parent strain (Table 1), further suggesting the deletion of *tmk3* leads to hypersensitivity to CFW and CR and reduction of cell wall integrity.

**Table 1:** Diameters of *T. reesei* parent strain and *T. reesei Δtmk3* colonies on CR- and CFW- containing PDA plates.

CR-containing plates				
CR concentration (mg/L)	*T. reesei* parent strain colony diameter (mm)	Percentage of reduction (*versus* no CR addition)	*T. reesei Δtmk3* colony diameter (mm)	Percentage of reduction (*versus* no CR addition)
0	48.3±0.6	0.0%	32.0±0.0	0.0%
75	33.0±0.0	31.7%	18.5±0.7	42.2%
125	28.7±0.6	40.7%	0.0±0.0	100.0%
150	25.3±0.6	47.6%	0.0±0.0	100.0%
175	22.0±1.0	54.5%	0.0±0.0	100.0%
200	18.0±1.0	62.8%	0.0±0.0	100.0%

CFW-containing plates				
CFW concentration (mg/L)	*T. reesei* parent strain colony diameter (mm)	Percentage of reduction (*versus* no CFW addition)	*T. reesei Δtmk3* colony diameter (mm)	Percentage of reduction (*versus* no CFW addition)
0	50.3±1.5	0.0%	42.0±0.0	0.0%
10	47.3±0.6	6.0%	32.5±0.7	22.6%
20	24.7±0.6	51.0%	16.5±0.7	60.7%
30	22.3±0.6	55.6%	0.0±0.0	100.0%
40	14.3±1.2	71.5%	0.0±0.0	100.0%

**Figure 5:**
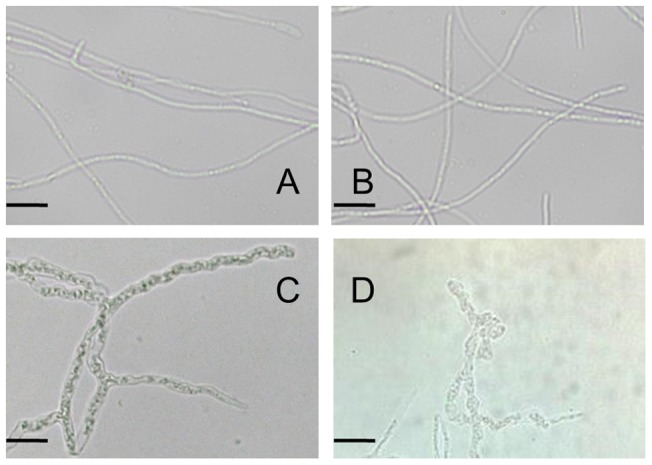
Microscopic images of *T. reesei* parent strain and *T. reesei Δtmk3* hyphae. Panel A–B, *T. reesei* parent strain; Panel C–D, *T. reesei Δtmk3*. Bar, 20 μm.

Comparison of the hyphal phenotype of *T. reesei* parent and *Δtmk3* strains further supported the presence of weakened cell wall in the *tmk3* knockout strain. *T. reesei* parent strain hyphae have a smooth cell wall structure (Fig. 5A, 5B), while *T. reesei Δtmk3* hyphae apparently adopt a ‘budded’ appearance, in agreement with having compromised cell wall structure (Fig. 5C, 5D), although other possible defects such as impaired polarity maintenance cannot be excluded [48], [49].

**Figure 6:**
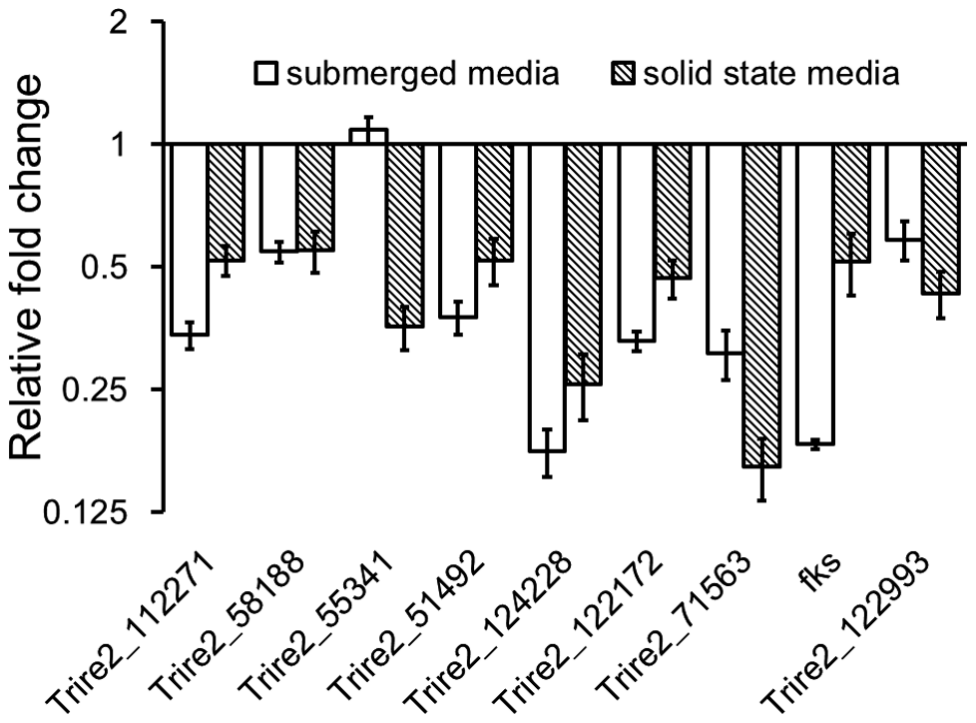
Transcriptional changes of chitin synthase- and β-1,3-glucan synthase-coding genes. Y-axis indicates transcriptional abundance ratio between *T. reesei Δtmk3*/*T. reesei* parent strain. Trire2_112271, Trire2_58188, Trire2_55341, Trire2_51492, Trire2_124228, Trire2_122172, Trire2_71563, Trire2_67600, chitin synthase-coding genes; *fks*, β-1,3-glucan synthase-coding genes.

The fungal cell wall is primarily composed of proteins and polysaccharides including chitin, β-1,3-glucan and β-1,6-glucan [50]. It has been previously reported that chitin synthase and β-1,3-glucan synthase are involved in the synthesis of chitin and β-1,3-glucan in filamentous fungi [51], [52]. Nine chitin synthase coding genes (Trire2_112271, Trire2_58188, Trire2_55341, Trire2_51492, Trire2_124228, Trire2_122172, Trire2_71563, Trire2_67600) and one β-1,3-glucan synthase coding gene (Trire2_78176, *fks*) are present in the genome of *T. reesei*. The transcriptional abundance of these genes in *T. reesei* parent and *Δtmk3* strains was investigated with real-time PCR. The transcription of all these genes except for Trire2_67600 was detected, and all the detected genes except for Trire2_55341 are significantly (2∼8 folds) downregulated in *T. reesei Δtmk3* (Fig. 6), suggesting Tmk3 participates in the cell wall integrity pathway by regulating the synthesis of chitin and β-1,3-glucan. These transcriptional responses were further supported by the observation of higher chitin content in *T. reesei* parent strain (4.19±0.07 percent DCW) than in *T. reesei Δtmk3* strain (3.48±0.07 percent DCW).

Our results reported in this study clearly suggest the involvement of Tmk3 in the maintenance of cell wall integrity in *T. reesei*. Interestingly, instead of Hog1-type MAPKs, Slt2-type MAPKs were reportedly involved in cell wall integrity maintenance (as is summarized in the introduction section). The unaltered or even increased resistance to CR and CFW in *hog1* deletion strains of *T. harzianum* and *A. fumigatus* further suggested Hog1 is not involved in cell wall integrity pathways in these two filamentous fungi [21], [27]. The ‘budded’ appearance of *T. reesei Δtmk3* hyphae in minimal media is similar to that of *A. fumigatus ΔmpkA* (MpkA is a Slt2 homologue in *A. fumigatus*) [15], while this phenomenon was only apparent in *hog1* deletion strains of *M. grisea* and *A. nidulans* when exposed in high salt media due to their incapability to cope with high osmotic pressure [10], [19]. All these observations lead to the suggestion that Tmk3, unlike Hog1-type MAPKs in other fungi, functions in the cell wall integrity pathway similarly to Slt2-type MAPKs. This also explains the discrepancy of growth performance in standard low salt media between *T. reesei Δtmk3* and other filamentous fungi in which *hog1* homologues were inactivated [10], [13], [21]: Hog1 in other filamentous fungi only responds to high-salt environments, so they grow normally in low salt media; Tmk3 is involved in cell wall integrity maintenance, and there is a growth defect for *T. reesei Δtmk3* even under low osmolarity.

### Responses of cellulase and hemicellulase production to *tmk3* deletion

**Figure 7:**
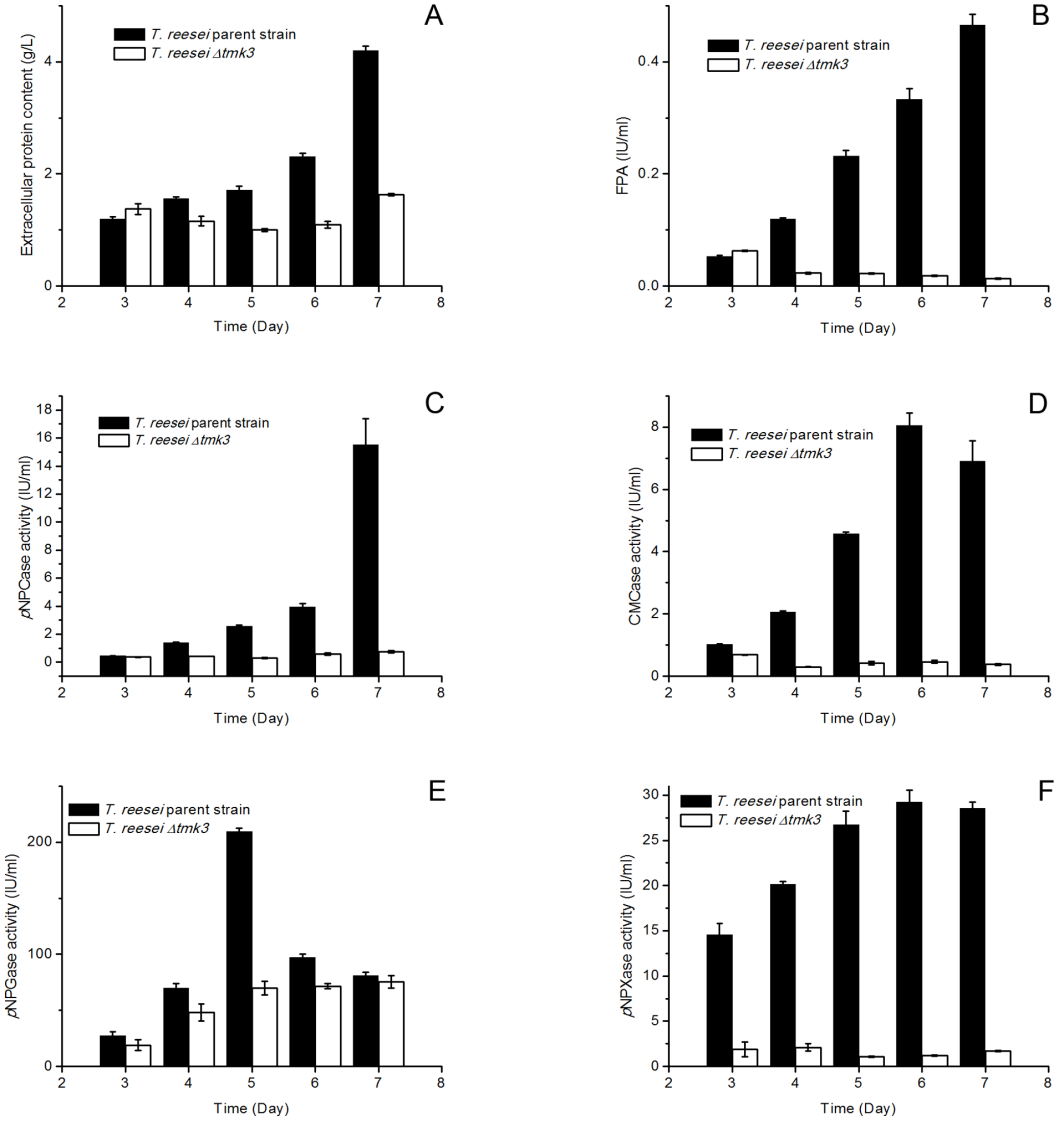
Production of extracellular proteins, cellulases and hemicellulase in submerged media. Panel A, Extracellular protein levels; panel B, FPA levels; Panel C, *p*NPCase activities; Panel D, CMCase activities; Panel E, *p*NPGase activities; Panel F, *p*NPXase activities.

**Figure 8:**
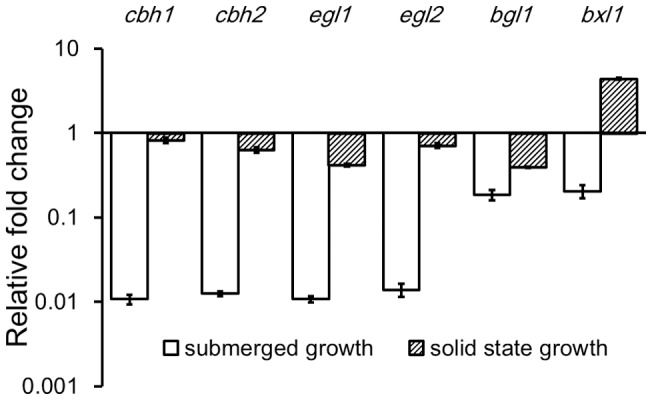
Transcriptional changes of cellulase- and hemicellulase-coding genes. Y-axis indicates transcriptional abundance ratio between *T. reesei Δtmk3* and *T. reesei* parent strain.

The comparison of cellulase production from *T. reesei* parent and *Δtmk3* strains under submerged cultivation conditions showed significantly decreased production of FPA, extracellular protein, *p*NPCase activity that measures cellobiohydrolase activity, CMCase activity that measures endoglucanase activity, *p*NPGase activity that measures β-glucosidase activity, and *p*NPXase activity that measures β-xylosidase activity in *T. reesei Δtmk3* (Fig. 7). Transcriptional analysis showed significant and strong downregulation of *cbh1*, *cbh2*, *egl1*, *egl2*, *bgl1* and *bxl1*, respectively coding CBHI, cellobiohydrolase II (CBHII), endoglucanase I (EGI), endoglucanase II (EGII), β-glucosidase I (BGI) and β-xylosidase I (BXL1) (Fig. 8).

The decrease in cellulase and hemicellulase production in *T. reesei Δtmk3* cannot be solely explained by the reduced biomass accumulation, because the transcriptional levels of cellulase- and hemicellulase-coding genes are clearly downregulated. These observations lead to the suggestion that Tmk3 is involved in cellulase production and/or induction. In *T. reesei*, several transcription factors were shown to regulate cellulase production, including the activator Xyr1 [53], repressor Cre1 [54], repressor ACEI [55] and activator ACEII [56]. Using a phosphorylation prediction software KinasePhos [57], we were able to identify MAPK phosphorylation sites on all these transcription factors (Table 2). Previous reports have suggested that the phosphorylation of some of these transcription factors is the prerequisite for their activities [58], [59]. It is therefore reasonable to propose that Tmk3 may regulate cellulase production by phosphorylating, and therefore activating, transcription factors. This proposal, however, should not be interpreted as the activation of ALL these transcription factors by Tmk3, as two more MAPKs (Tmk1, Tmk2) are present in *T. reesei*, and the phosphorylation specificity of the transcription factors has not been identified.

**Table 2:** Predicted MAPK phosphorylation sites in transcription factors.

Transcription factor	MAPK phosphorylation sites
Cre1	S164, S262, T281, T285, T289
Xyr1	S324, T405, T443
ACEI	S10, S42, T365, T393, T513, T521, S523, S524, T525, T614, T660
ACEII	T201

### Restored growth and cellulase production in solid state media

One common problem on the studies of *T. reesei* physiology is that the way *T. reesei* is cultured in these investigations does not resemble their natural habitats. In nature, *T. reesei* grows on solid lignocellulosic particles, instead of in submerged media which are normally used for *T. reesei* cultivation during physiological studies. There are considerable differences between these two environments: the level of encountered moisture varies significantly; the exposure to oxygen is different; and in the natural environment, secreted proteins do not diffuse like during submerged cultivation. We therefore compared the performance of *T. reesei Δtmk3* in submerged media and solid state media, in order to further identify the role of Tmk3 in *T. reesei*.

**Figure 9:**
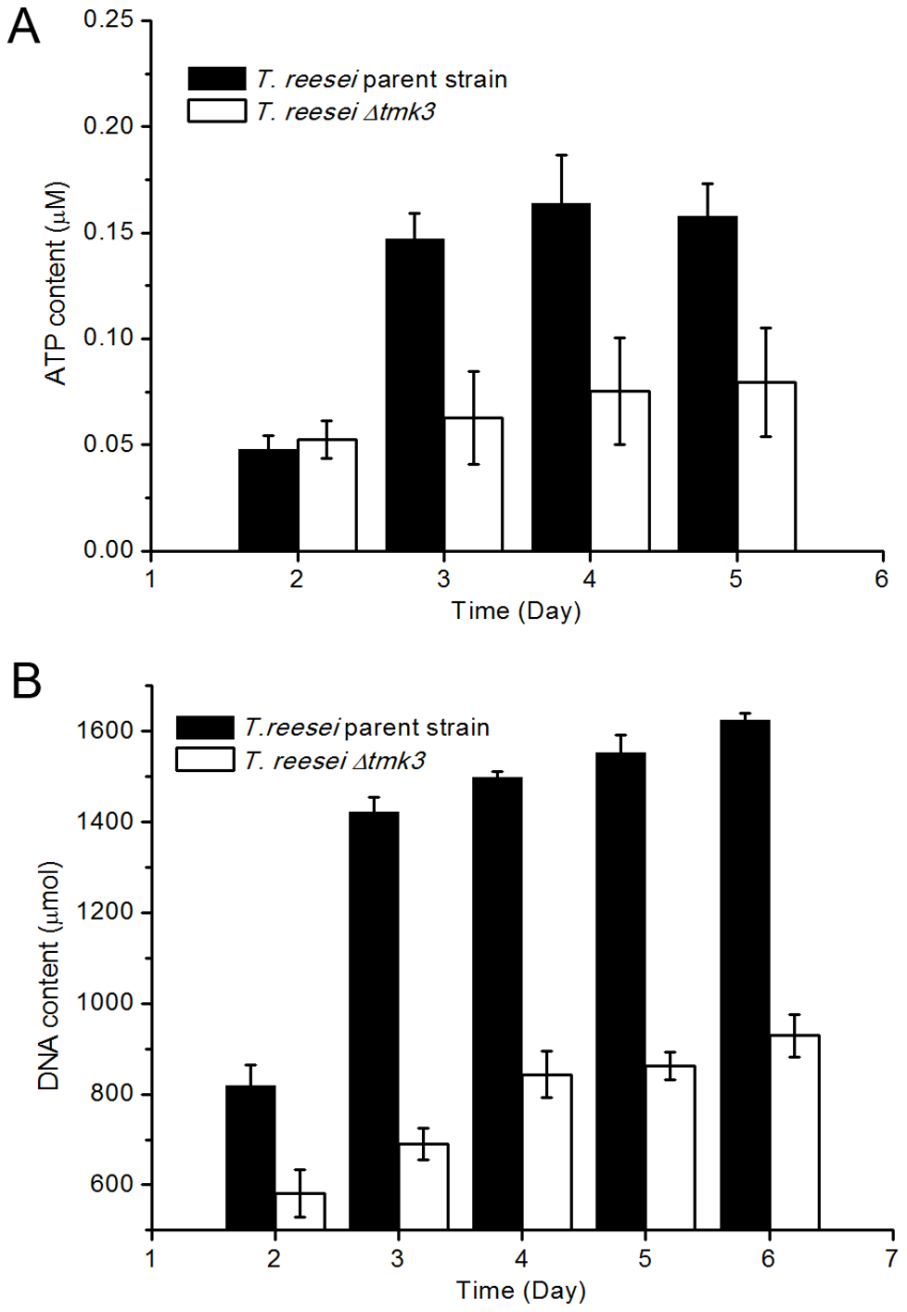
Biomass accumulations on solid state media. Panel A, biomass measured by ATP concentration in cultures; Panel B, biomass measured by total DNA content in cultures.

A significant improvement in ATP and DNA contents, as measures of biomass accumulation, could be observed when *T. reesei Δtmk3* was grown on solid state media (Fig. 9), when compared to submerged cultures (Fig. 2). However, in comparison with submerged cultivation, chitin synthase and β-1,3-glucan synthase transcription showed a similar magnitude of downregulation during solid state cultivation following *tmk3* deletion, except for Trire2_55341 (Fig. 6). Therefore, the improved ATP and DNA contents, as measures of biomass production, during solid state cultivation for *T. reesei Δtmk3* can unlikely be attributed to recovered cell wall integrity. It has been known for a long time that cell walls serve as the skeletons of fungal cells, and that fungal protoplasts die due to inflow of water unless osmolarity in the growth medium is adjusted [60]. One proposed explanation to the observed improvement in biomass accumulation for *T. reesei Δtmk3* is that during solid state cultivation, secreted proteins do not diffuse away from the fungal cells, therefore increasing regional protein accumulation and subsequently regional osmolarity. This ‘protein layer’ could serve as the osmotic stabilizer for fungal cells with weakened cell wall, and could lead to improved growth of *T. reesei Δtmk3* in solid-state media.

In contrary to chitin synthase and β-1,3-glucan synthase encoding genes, cellulase and hemicellulase encoding genes show a significantly different transcription pattern in *T. reesei Δtmk3*. The transcription of *cbh1*, *cbh2*, *egl1*, *egl2*, *bgl1* was downregulated by 1.2 to 2.6 fold during solid state cultivation (Fig. 8), while the magnitude of downregulation was respectively 93, 80, 93, 73 and 5.5 fold during submerged cultivation. The transcription of *bxl1* was downregulated by 4.9 fold during submerged growth, but was upregulated by 4.4 fold during solid state growth. The behavior of *bgl1* and *bxl1* transcription was not the same as that of *cbh1*, *cbh2*, *egl1* and *egl2*, suggesting they were regulated differently.

**Figure 10:**
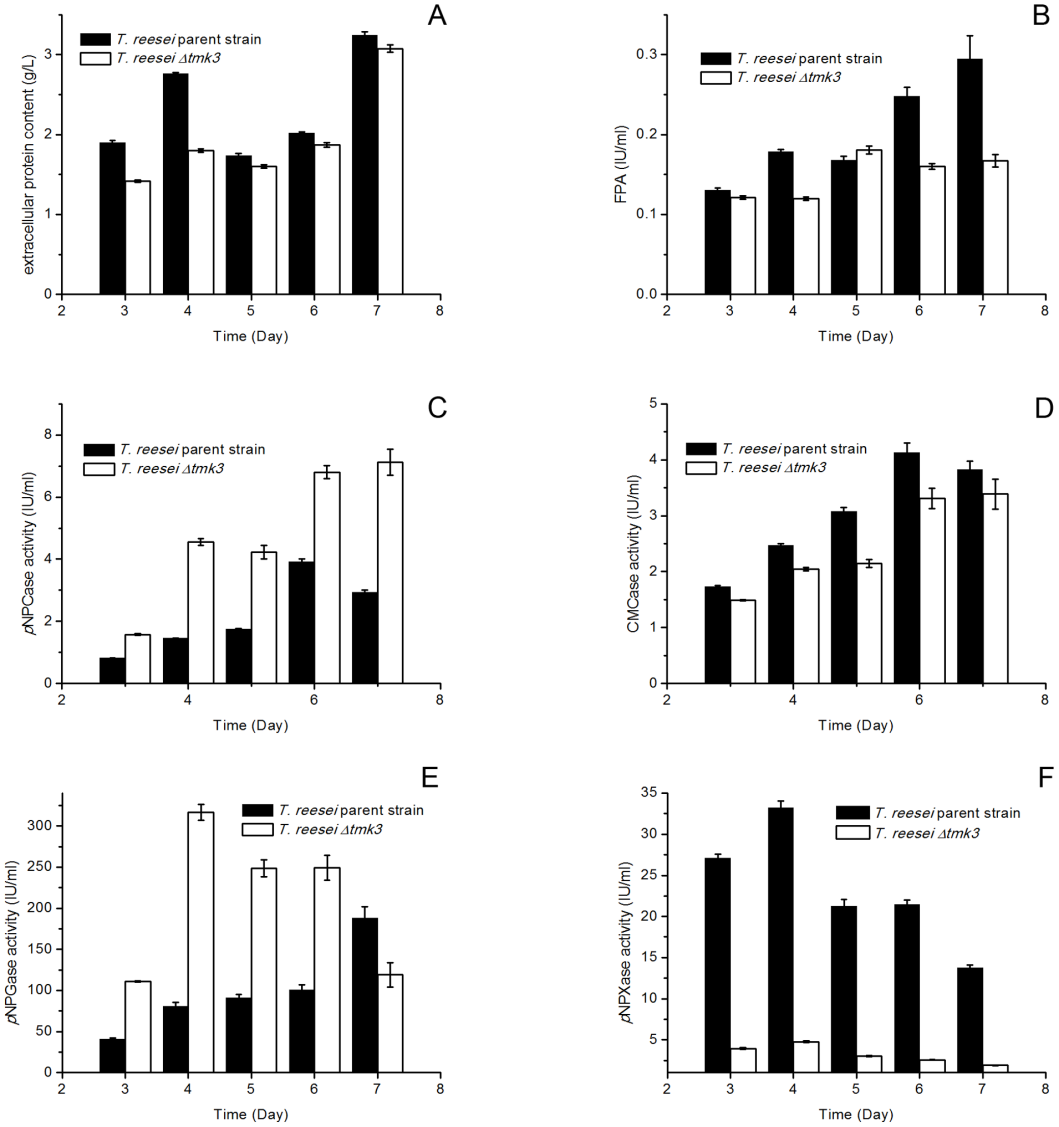
Production of extracellular proteins, cellulases and hemicellulase in solid state media. Panel A, Extracellular protein levels; panel B, FPA levels; Panel C, *p*NPCase activities; Panel D, CMCase activities; Panel E, *p*NPGase activities; Panel F, *p*NPXase activities.

Extracellular protein level and cellulase activities in *T. reesei Δtmk3* cultures were partially restored during solid-state cultivation similar to the transcriptional abundance (Fig. 10A–E). β-xylosidase activity was not restored, although the transcription of *bxl1* was upregulated in the *tmk3* deletion strain (Fig. 10F), again suggesting β-xylosidase production is regulated differently from cellulases. Interestingly, the production of *p*NPCase and *p*NPGase activities is improved in *T. reesei Δtmk3* when comparing with the parent strain (Fig. 10C, E). This is consistent with our observation that a larger clearing zone was present around *T. reesei Δtmk3* colonies than *T. reesei* parent strain colonies when they are grown on double-layer avicel plates, because the surrounding environment of aerial hyphae on plates is similar to solid state cultivation. One proposed explanation to this improvement of cellulase production is that the weaker cell wall in *T. reesei Δtmk3* allows easier protein permeation and therefore may benefit secretion and/or release of cell bound enzyme or protein. This beneficial effect could not be offset by the slight downregulation of *cbh1* and *bgl1* transcription and decreased biomass accumulation, and therefore leads to the improvement of overall cellobiohydrolase and β-glucosidase production.

Providing an explanation for the restoration of transcription for all the cellulase-coding genes during solid state cultivation is difficult before investigations could provide further evidence for the involvement of an additional regulatory mechanism. However, these results do suggest the interplay between Tmk3 mediated and other cellulase/hemicellulase regulatory mechanisms. Indeed, cellulase/hemicellulase production is regulated by a complicated regulatory network that is governed by many signals (such as light, carbon source etc), as well as extracellular and cytoplasmic factors [34], [36]. It is therefore not a surprise for Tmk3 mediated regulatory mechanism and other regulatory mechanisms to affect each other.

Our studies of *T. reesei Δtmk3* grown during solid state cultivation showed a clear restorative effect in comparison to submerged cultivation. The molecular mechanisms underlying these effects vary on different aspects of cellular physiology, but the unified final restorative effect suggests *T. reesei* physiology is evolved to favor solid state cultivation. Although secreted proteins or metabolites can be produced more efficiently from filamentous fungi during submerged cultivation, therefore benefiting industrial application, the submerged condition might actually alter fungal growth and metabolism, and care needs to be taken when assumptions are made that growth under certain submerged growth conditions is ‘optimum’ or ‘physiological’, particularly during mechanistic studies on fungal physiology.

## Conclusions

Results reported herein lead to the suggestion of the physiological roles in high osmolarity resistance, cell wall integrity maintenance and cellulase production of a Hog1-type MAPK Tmk3 in *T. reesei*. Although sequence comparison suggests Tmk3 is homologous to Hog1p from *S. cerevisiae*, their functions appear to vary significantly. While Hog1p from *S. cerevisiae* and Tmk3 from *T. reesei* are both involved in the resistance to high osmolarity, the mechanism of this function differs: induction of osmotic stabilizer resistance genes in *S. cerevisiae* and derepression of these genes in *T. reesei*. Further investigations showed the function of Tmk3 in more novel aspects. Our phenotypic analysis, chemical sensitivity studies and transcriptional profiling all suggested Tmk3 functions in the cell wall integrity signaling pathway similarly to Slt2-type MAPKs, a role never identified for Hog1-type MAPK in filamentous fungi before. We further observed that deletion of *tmk3* leads to an apparent decrease in cellulase transcription, and suggested the involvement of Tmk3 in cellulase production regulation by phosphorylating transcription factors.

When grown in submerged media, biomass accumulation and cellulase production of *T. reesei* were significantly reduced upon *tmk3* deletion. The degree of reduction was much smaller after transferring to solid-state media. The mechanism of the restoration of growth likely differs from the mechanism of the restoration of cellulase production, but the similarity between the effects suggests *T. reesei* is evolved to favor solid state cultivation.

In conclusion, our results show the participation of Tmk3 in high osmolarity resistance and two novel aspects: cell wall integrity and cellulase production regulation. The restorative effect identified during solid cultivation, particularly regulation of cellulase production, is worthy of further investigations for the identification of other regulatory pathways involved in cellulase production.

## Supporting Information

Figure S1 Phylogenetic analysis of MAPKs from filamentous fungi. The phylogenetic tree was constructed by the neighbour-joining method. Bootstrap values are shown at each note, and are calculated from 1000 trees. Bar, evolutionary distance of 0.1. Thhog1, Hog1 from *Trichoderma harzianum*;Trtmk3, Tmk3 from *Trichoderma reesei*; Ncos2, Os-2 from *Neurospora crassa*; Cpmk1, Mk1 from *Cryphonectria parasitica*; AfsakA, SakA from *Aspergillus fumitagus*; AnhogA, HogA from *Aspergillus nidulans*; TvtmkB, TmkB from *Trichoderma virens*; Trtmk2, Tmk2 from *T. reesei*; Fgmgv1, Mgv1 from *Fusarium graminearum*; Bcbmp3, Bmp3 from *Botrytis cinerea*; Cpmk2, MK2 from *Claviceps purpurea*; AfmpkA, MpkA from *A. fumigatus*; TvtmkA, TmkA from *T. virens*; Tvtvk1, Tvk1 from *T. virens*; Trtmk1, Tmk1 from *T. reesei*; Tatmk1, Tmk1 from *Trichodera atroviride*.
(TIF)

Figure S2 Sequence alignment of Tmk3 from *T. reesei* and Hog1 from *Saccharomyces cerevisiae*. Trtmk3, Tmk3 from *T. reesei*; Schog1, Hog1 from *S. cerevisiae*.
(TIF)

Figure S3 Southern blotting analysis of *T. reesei* parent and *Δtmk3* strains. M, DNA molecular size marker; *Δtmk3*, *T. reesei Δtmk3*; parent, *T. reesei* parent strain. Indicated by arrows are the predicted sizes of DNA fragments hybridized with the probe.
(TIF)
