# Supplementary figures and images for: A Mitogen-Activated Protein Kinase Tmk3 Participates in High Osmolarity Resistance, Cell Wall Integrity Maintenance and Cellulase Production Regulation in Trichoderma reesei
Source: PLoS One. 2013 Aug 26;8(8):e72189. doi: 10.1371/journal.pone.0072189 (PMC3753334; doi:10.1371/journal.pone.0072189)

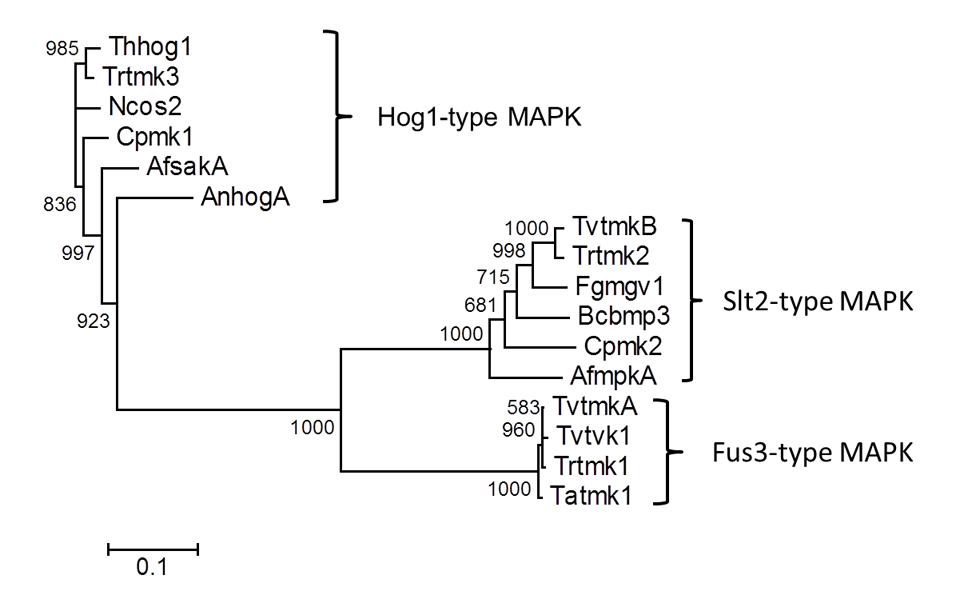

Supplement: Figure S1 — Phylogenetic analysis of MAPKs from filamentous fungi. The phylogenetic tree was constructed by the neighbour-joining method. Bootstrap values are shown at each note, and are calculated from 1000 trees. Bar, evolutionary distance of 0.1. Thhog1, Hog1 from Trichoderma harzianum;Trtmk3, Tmk3 from Trichoderma reesei; Ncos2, Os-2 from Neurospora crassa; Cpmk1, Mk1 from Cryphonectria parasitica; AfsakA, SakA from Aspergillus fumitagus; AnhogA, HogA from Aspergillus nidulans; TvtmkB, TmkB from Trichoderma virens; Trtmk2, Tmk2 from T. reesei; Fgmgv1, Mgv1 from Fusarium graminearum; Bcbmp3, Bmp3 from Botrytis cinerea; Cpmk2, MK2 from Claviceps purpurea; AfmpkA, MpkA from A. fumigatus; TvtmkA, TmkA from T. virens; Tvtvk1, Tvk1 from T. virens; Trtmk1, Tmk1 from T. reesei; Tatmk1, Tmk1 from Trichodera atroviride. (TIF) [file pone.0072189.s001.tif]

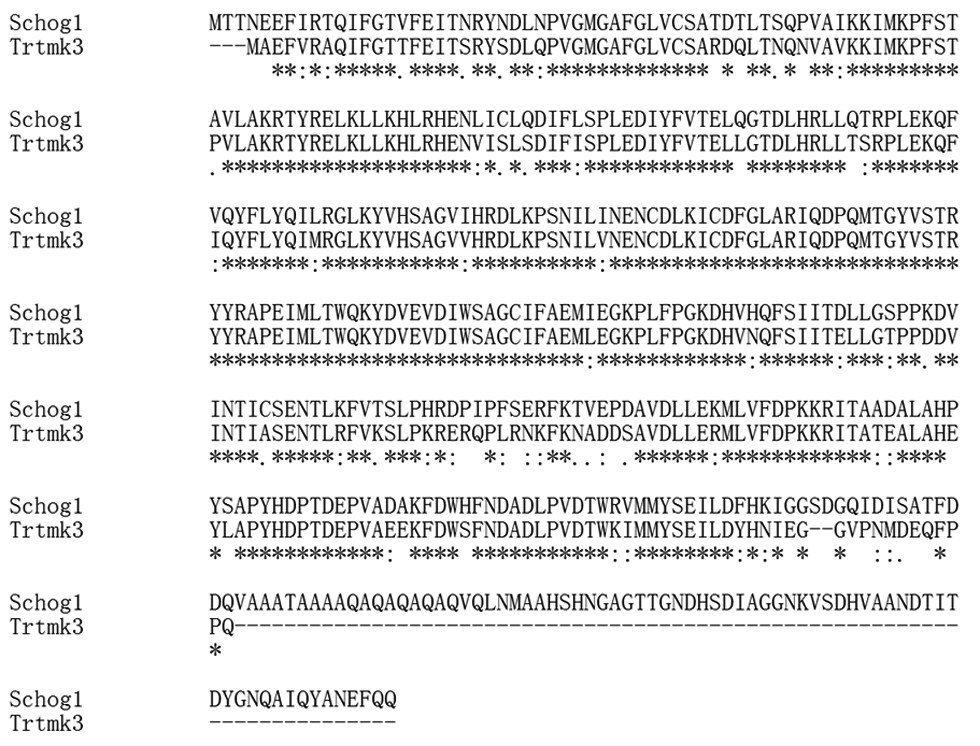

Supplement: Figure S2 — Sequence alignment of Tmk3 from T. reesei and Hog1 from Saccharomyces cerevisiae. Trtmk3, Tmk3 from T. reesei; Schog1, Hog1 from S. cerevisiae. (TIF) [file pone.0072189.s002.tif]

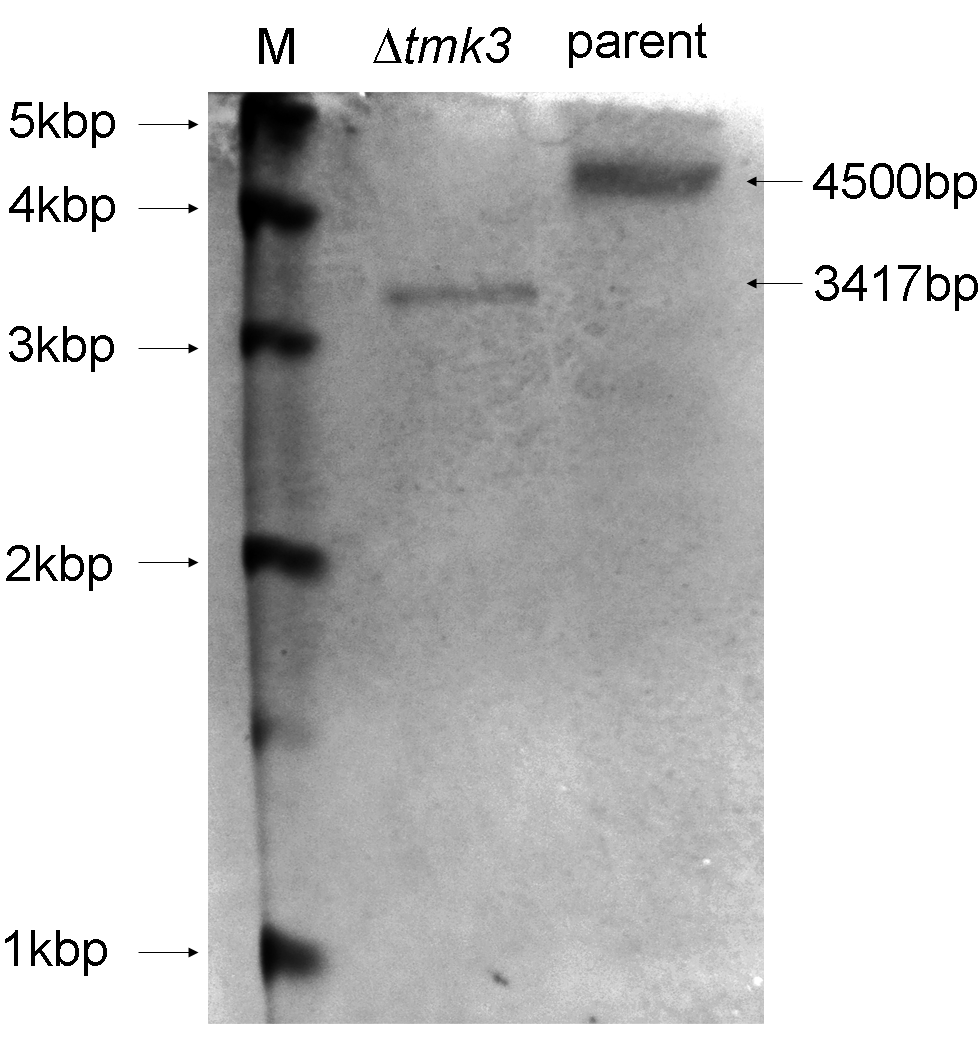

Supplement: Figure S3 — Southern blotting analysis of T. reesei parent and Δtmk3 strains. M, DNA molecular size marker; Δtmk3, T. reesei Δtmk3; parent, T. reesei parent strain. Indicated by arrows are the predicted sizes of DNA fragments hybridized with the probe. (TIF) [file pone.0072189.s003.tif]
